# Assessment of parasitic pollution in the coastal seawater of Gaza city

**DOI:** 10.1186/2052-336X-12-26

**Published:** 2014-01-10

**Authors:** Ahmed Hisham Hilles, Adnan Ibrahim Al Hindi, Yousef Attalla Abu Safieh

**Affiliations:** 1 Institute of Water and Environment, Al Azhar University, Gaza, Palestine; 2 Medical Laboratory Sciences Department, Faculty of Health Sciences, Islamic University of Gaza, P.O. Box 108, Gaza, Palestine; 3 Minister, Ministry of Environmental Affairs, Palestinian National Authority, Gaza, Palestine

**Keywords:** Assessment, Parasitic contamination, Shoreline region, Wastewater and carrying currents

## Abstract

**Background:**

The main objective of the study was the assessment of the prevalence and the identification of species of human gastrointestinal parasites as an indicator of the pollution of the seashore of Gaza City.

**Methods:**

The investigation was conducted by analysis of the parasitic contamination of seawater along the study area. A total of 52 samples of seawater were analyzed during the summer period; from June to October 2011. The study area was divided into six zones (A, B, C, D, E and F) according to specific criteria such as the presence of the wastewater discharge points and other geographical characteristics.

**Results:**

The results show that about 48% of the seawater samples from the shoreline region of Gaza City were contaminated with parasites. Zones A, B and D (mouth of Wadi Gaza, Al Sheikh Ejleen discharge and Al Shalehat discharge points respectively) have the highest level of parasitic contamination, while, zones C and E (From Al-Baydar restaurant to Khalel Alwazer Mosque and the basin of the Gaza marina respectively) had a lower level of contamination and zone F (From the northern part of the Gaza marina to the Intelligence Building) was uncontaminated. The parasitic species found were: *Ascaris lumbricoides, Giardia lamblia, Strongyloides stercoralis, Hymenolepis nana, Entamoeba histolytica/dispar* and *Cryptosporidium parvum.*

**Conclusions:**

The present study revealed a high level of contamination with parasites at most of the points which were investigated along the Gaza City coast line.

## Introduction

**Figure 1:**
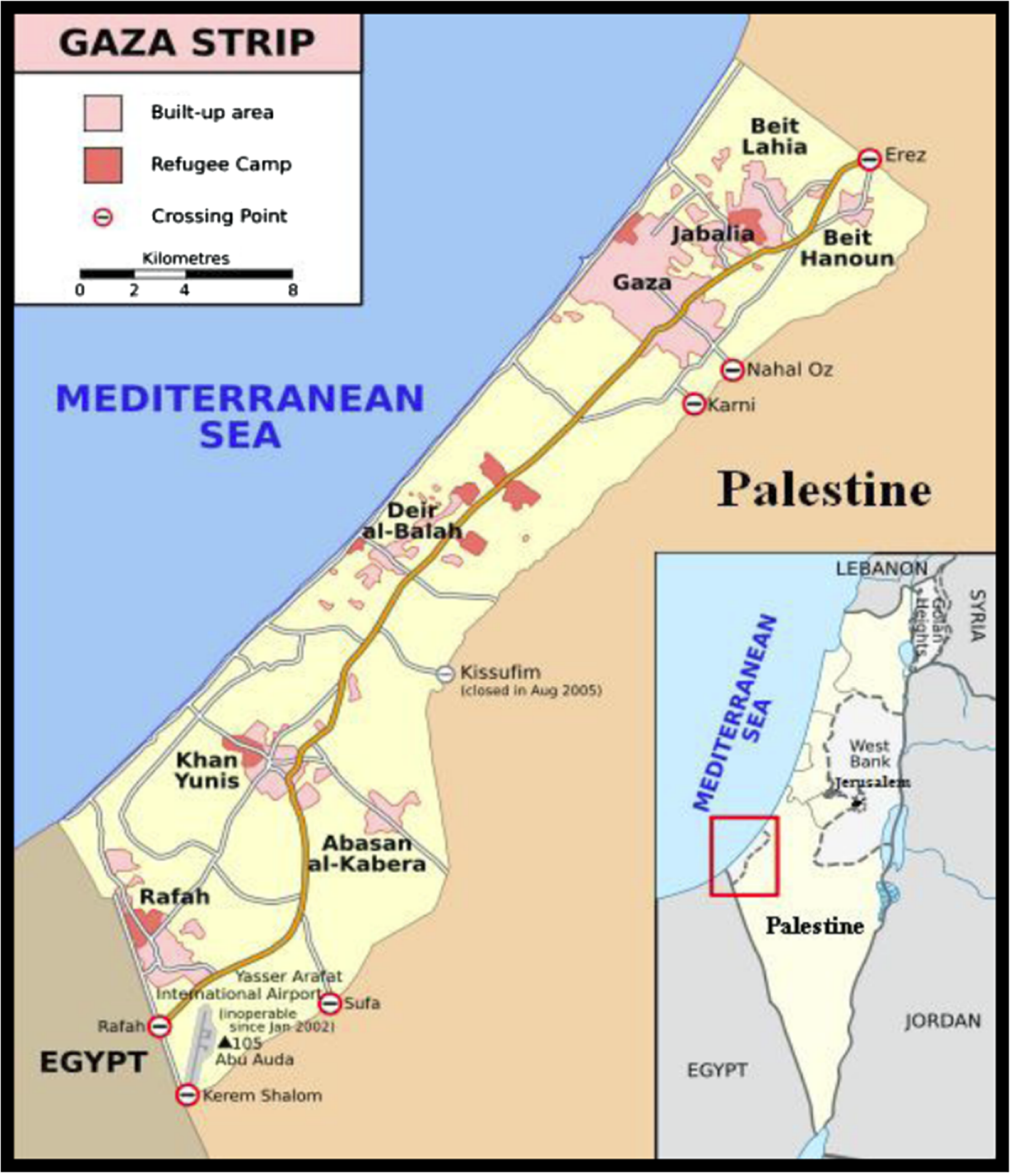
Gaza Strip as part of Palestine.

The Gaza Strip is located on the south east corner of the Mediterranean Sea. The length of the Gaza Strip shore on the Mediterranean is about 41 km. The width of the strip ranges between 5 km in the middle to 8 km in the north and 12 km in the south as shown in Figure 1. The Gaza Strip is bounded by the green line with Israel from north and east and by Egypt from the south and by the Mediterranean in the west. The total population is about 1.6 million inhabiting a total area of 378 km^2^[1,2].

Most of the wastewater treatment plants (WWTPs) in Gaza are overloaded and are working beyond their designed capacities [3]. According to the Coastal Municipalities Water Utility (CMWU), 2011, about 89 million liters per day (MLD) of untreated or partially treated sewage is discharged into the sea, made up of 69MLD of partially treated sewage and 20MLD of raw sewage. The lack of proper wastewater treatment facilities leads to the discharge of untreated or partially treated sewage directly to the seashore and indirectly through Wadi Gaza from the middle camps (Nuseirat, Bureij, Maghazi) which finally reach the sea. Many discharge points are registered along the shoreline in the Gaza Strip [4]. Microbiologically contaminated seawater has been found along the Gaza Strip coast [5], and there is an evidence of sanitation-related infections in the Gaza Strip [6-8]. The 41 km of shoreline is already under intense pressure, with substantial environmental degradation of terrestrial and marine resources [9]. Recreational use of water and beaches is an important feature of leisure and tourism worldwide. There is also an important interaction between tourism and the environment. A healthy attractive environment is one of the principal considerations in selecting a holiday destination [10].

Seawater and beach quality monitoring and assessment are considered as vital parts of any integrated coastal management, and extensive research with the aim of establishing guidelines and standards for recreational water quality has been conducted all over the world [11]. Intestinal parasitic infection in the Gaza Strip is still a problem; this is probably due to poor general health, poor sanitation, high population density, bad hygiene habits and poor health education [12]. Several studies carried out in the Gaza Strip on the prevalence of parasitic diseases among school children revealed a prevalence of intestinal parasitic infections in children aged 6–12 years old from Deir El-Balah of 36.3% and of 72.9% in children from Beit-lahia [13,14]. The aim of this study was to assess the level of Parasitic Contamination due to Wastewater Discharge in the seawater of Gaza City. The Specific Objectives were: to Specify and identify the parasitic contamination (species and percentages) in the shoreline region of Gaza City, to determine the source of the parasitic pollution in the sea water, to determine the zone vulnerable to parasitic contamination and identify the level of vulnerability and to determine the effect of the carrying currents on the distribution of parasites along the shoreline region of Gaza City.

## Material and methods

### Study location

**Figure 2:**
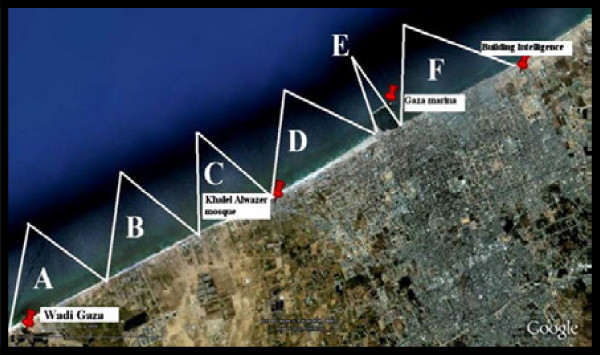
**Study area and the zones of sampling from A to F**[15]**.**

The study area (about 12 km of the Gaza City shoreline) was divided into six sampling zones in order to facilitate the sampling process as shown in Table 1. The study area was divided into these zones (Figure 2) according to factors such as; location from sewage discharge points (outlets), tourist and/or recreational features and the distance between zones.

**Table 1:** Zones of sampling and related information

**Zone symbol**	**Zone boundaries**	**Zone length**	**Total number of samples**
**A**	From Wadi Gaza to Al-Zahra City	1800 m	(12 samples)*
			-First 5 samples every 50 m.
			-Second 5 samples every 250 m.
**B**	Al-Zahra City to Al-Baydar restaurant	2000 m	**(5 samples)**
			-Every 500 m
**C**	From Al-Byder restaurant to Khalel Alwazer mosque	1800 m	**(6 samples)**
			- Sample every 300 m.
**D**	From Khalel Alwazer mosque to the southern part of the Gaza marina	2500 m	**(12 samples)**
			- Sample every 200 m.
**E**	The basin of the Gaza marina	450 m	**(4 samples)**
			-Every 100 m.
**F**	From the northern part of the Gaza marina to the intelligence building	2800 m	**(13 samples)**
			- Sample every 200
**Total**	**Study area**	**12 km**	**52 samples**

*Two samples of the 12 samples were collected from the southern part (300 m to the south) of Wadi Gaza discharge point.

The following map in Figure 1, clearly shows the selected zones on Gaza City seashore.

### Study duration

This study was carried out over a 12-month period from March, 2011 to March, 2012. Sampling processes were conducted throughout the summer season months (from June to October/2011).

### Sample size

A total of 52 samples were collected within several sampling rounds from the different zones (A to F) in order to be representative and generalize the results of the study as shown in Table 1 and Figure 2.

### Sample collection

Samples were collected from the above mentioned six zones. From each one of these six collection zones, a number of collection sites were regularly defined. Samples of seawater were collected from systematic and calculated distances in each zone (Table 1). These zones as a whole represent the entire shoreline region of the Gaza City.

### Seawater sampling procedure

Using a horse and cart, seawater samples were collected in sterile 4 L (4000 ml) plastic bottles according to the APHA, 1995 standard methods. Seawater samples were transferred to the laboratory and processed within 24 h of collection [16].

### Seawater analysis

In order to analyze seawater samples and to identify the parasitic pollution, three techniques were applied:

A. Direct Seawater Smear Microscopy (Wet Mount)

In this technique, samples of the seawater were directly examined under the microscope without any pretreatment [17].

B. Seawater Concentration Technique

This technique was applied through the following steps: A sample of 4 L of seawater was filtered using a Buchner funnel and membrane filters of 7 to 10 μm pores connected to a side-arm flask by means of a neoprene adapter, with a tube leading to a vacuum pump. The sample was filtered but without drying the filter by discontinuing the suction. The filter paper was transferred to a side wall of a 100 ml beaker, and repeatedly flushed with several milliliters of sterile distilled water [18]. The solution obtained after washing was Centrifuged (2000 rpm) for 10 min using a 15 ml centrifuge tube [19]. The supernatant was discarded and the sediment was collected. One drop of the sediment was placed in the center of a slide. The drop was covered with a cover slip by holding the cover slip at an angle, touching the edge of the drop, and gently lowering the coverslip onto the slide so that air bubbles are not produced. The slide was examined as recently illustrated. The sediment stored in a labeled Opened rove tube (with sharp bottom and snap cap) for staining and photography.

C. Staining Technique

In the present study, Ziehl-Neelsen/Acid-Fast Stain was used to detect *C. parvum*[19].

### Data entry and analysis

Statistical Package for the Social Science program (SPSS) version 18 was used for data analysis. Descriptive statistics in term of frequencies, means, percentage, standard deviation, independent sample T.test, ANOVA and Correlation Pearson were used for the different variables.

## Results

The parasite species that have been detected in the seawater using the three examination techniques (direct smear, concentration and staining) are illustrated in Table 2.

**Table 2:** Species of parasites detected in seawater using the three techniques

**No.**	**Zone**	**Code**	**Direct smear**	**Concentration technique**	**Staining technique**
1	**A**	A-1*	N	N	N
2		A-2*	N	N	N
3		A1	*- S. stercoralis*	*- A.lumbricoides*	*C. parvum*
				*–H. nana*	
				*-E. histolytica*	
4		A2	*- S. stercoralis*	*- A.s lumbricoides*	*C. parvum*
5		A3	N	*- A. lumbricoides*	*C. parvum*
				*- S. stercoralis*	
6		A4	N	*- A. lumbricoides*	N
7		A5	N	N	*C. parvum*
8		A6	N	N	*C. parvum*
9		A7	N	N	N
10		A8	N	N	N
11		A9	N	N	*C. parvum*
12		A10	N	N	*C. parvum*
13	**B**	B1	N	N	*C. parvum*
14		B2	N	*- G. lamblia*	*C. parvum*
15		B3	N	*- A. lumbricoides*	*C. parvum*
16		B4	*- S. stercoralis*	*- A. lumbricoides*	*C. parvum*
17		B5	N	*-E. histolytica*/dispar	*C. parvum*
18	**C**	C1	N	*- A. lumbricoides*	*C. parvum*
19		C2	N	N	N
20		C3	N	N	N
21		C4	N	N	N
22		C5	N	N	*C. parvum*
23		C6	N	N	*C. parvum*
24	**D**	D1	N	N	N
25		D2	N	N	*C. parvum*
26		D3	N	N	N
27		D4	N	N	*C. parvum*
28		D5	*- S. stercoralis*	N	N
29		D6	N	N	N
30		D7	N	*- S. stercoralis*	N
31		D8	N	N	N
32		D9	*- S. stercoralis*	N	N
33		D10	N	N	*C. parvum*
34		D11	N	*- S. stercoralis*	*C. parvum*
35		D12	*- S. stercoralis*	*- A. lumbricoides*	*C. parvum*
				*- S. stercoralis*	
36	**E**	E1	N	N	N
37		E2	N	N	N
38		E3	N	N	*C. parvum*
39		E4	N	N	N
40 to 52		**F**	N		

*Samples from the southern side of Wadi Gaza.

A-From Wadi Gaza to Al-Zahra City.

B-From Al-Zahra City to Al-Baydar restaurant.

C-From Al-Baydar restaurant to Khalel Alwazer mosque.

D-From Khalel Alwazer mosque to the southern part of the Gaza marina.

E-The basin of the Gaza marina.

F-From the northern part of the Gaza marina to the Intelligence Building.

N- Negative.

It's clear from Table 2 that zone (F) was uncontaminated by parasites.

### Comparison of seawater analysis according to techniques

Using the direct smear method, 11.5% of contamination was detected, with the concentration technique detecting 23.1% of contamination, while the highest level of detection of contamination of 40.4% was obtained using the staining technique.

According to Table 3, the results show that zone (B) has the highest level of contamination with 100% of samples polluted, followed by 66.6% in zone (A) and (D), 50% in zone (C), 25% in zone (E), and finally no parasitic pollution was detected in zone (F).

**Table 3:** Percentages of contamination in seawater according to zones

**Sea water**	**A**		**B**		**C**		**D**		**E**		**F**		**Total**	
	**No.**	**%**	**No.**	**%**	**No.**	**%**	**No.**	**%**	**No.**	**%**	**No.**	**%**	**No.**	**%**
**Contaminated**	8	66.6	5	100	3	50.0	8	66.6	1	25.0	0	0.0	25	48.1
**Uncontaminated**	4	33.4	0	0.0	3	50.0	4	33.4	3	75.0	13	100	27	51.9
**Total**	12	100.	5	100.	6	100.	12	100.	4	100.	13	100.	52	100.0

### Parasite distribution within the research area zones (A, B, C, D, E and F)

The distribution of the intestinal parasites in the different zones is as follows:

● Zone (B), 50% of the parasites was *C. parvum,* 20% *A. lumbricoides, 10*% *S. stercoralis,* 10% *E. histolytica/dispar* and 10% *G. lamblia.*

● Zone (C), 75% of the parasites was *C. parvum* and 25% *A. lumbricoides.*

● Zone (D), 41.7% of the parasites was *C. parvum,* 50% *S. stercoralis,* and 8.3% *A. lumbricoides.*

● Zone (E), almost 100% of the parasites was *C. parvum*, and

● Finally in zone (F), no parasites have been detected.

The total number of the parasites discovered in the seawater samples was 43, and the most common species was *C. parvum* with a prevalence of 48.8%. The second most common species of parasite was *S. stercoralis* with a prevalence of 23.3% followed by *A. lumbricoides* with a prevalence of 18.6%, then *E. histolytica/dispar*, *G. lamblia* and *H. nana* with 4.7%, 2.3% and 2.3% respectively of the total number of the detected species of human gastrointestinal parasites.

### Seawater analysis

Using the three analyzing techniques in analyzing seawater samples, the percentage of contamination throughout the entire study area is shown in Table 4.

**Table 4:** Percentage of contaminated samples using three analyzing techniques

		**Direct smear**		**Concentration**		**Staining**	
		**No.**	**(%)**	**No.**	**(%)**	**No.**	**(%)**
Contaminated samples	Single contamination	6		9		21	
	Mixed contamination	-		3		-	
	Total contaminated samples	6	11.5	12	23.1	21	40.4
Uncontaminated		46	88.5	40	76.9	31	59.6
Total samples		52	100.0	52	100.0	52	100.0

Using the direct smear analyzing technique, only 11.5% of the samples were contaminated with parasites, all of the samples were contaminated by single species of parasites, *Strongyloides stercoralis* (larva, adult female and male). The percentage of contaminated samples of seawater using the concentration technique for analysis was 23.1%, while 76.9 of the samples were uncontaminated.

**Figure 3:**
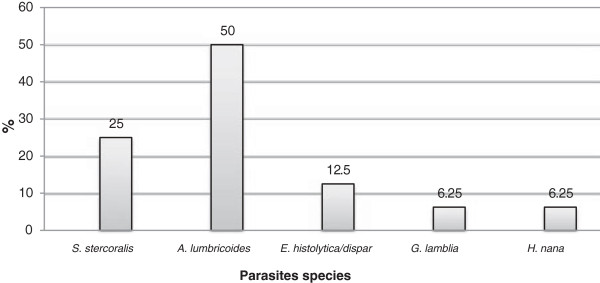
Detected parasite species and percentages in seawater using concentration technique.

The species and percentages of the parasites detected in the entire study area using the concentration technique are shown in Figure 3.

The above Figure 3 shows that the seawater samples using the concentration technique were contaminated with five species of human gastrointestinal parasites distributed as follows: 50% *A. lumbricoides,* 25% *S. stercoralis,* 12.5% *E. histolytica/dispar, and* 6.25% for both *G. lamblia* and *H. nana.*

### Total percentage of seawater contamination

By combining the three seawater analyzing techniques, the results of seawater contamination with gastrointestinal parasites are illustrated in Table 5. The table shows that 48.1% of the seawater samples from the entire study area (52 samples) were contaminated with parasites, with 51.9% of the total samples being uncontaminated. The number of seawater samples which were contaminated by one type of parasite using the three analyzing techniques was 15, and 10 of the seawater samples were contaminated by multiple human gastrointestinal parasite species.

**Table 5:** Total contamination percentage of seawater samples (by applying the three methods of analysis)

		**No.**	**(%)**
Contaminated	Single	15	
	Mixed	10	
	Total contaminated samples	25	48.1
Uncontaminated		27	51.9
Total samples		52	100.0

Table 6, illustrates the results of a single factor one way-ANOVA test for the spatial variation in the parasitic contamination within the six different zones (A, B, C, D, E, F) along the entire study area to examine whether there is a significant statistical difference in the contamination level through those zones and the level of significance. The results in the table indicate that there is a significant variation among the zones within the confidence level of a p-value of < 0.05).

**Table 6:** One way –ANOVA test for the parasitic pollution within the six zones

	**Sum of squares**	**df**	**Mean square**	**F**	**Sig.**
**Between groups**	11.133	5	2.227	13.011	.000
**Within groups**	43.467	254	.171		
**Total**	54.600	259			

### Carrying current

**Figure 4:**
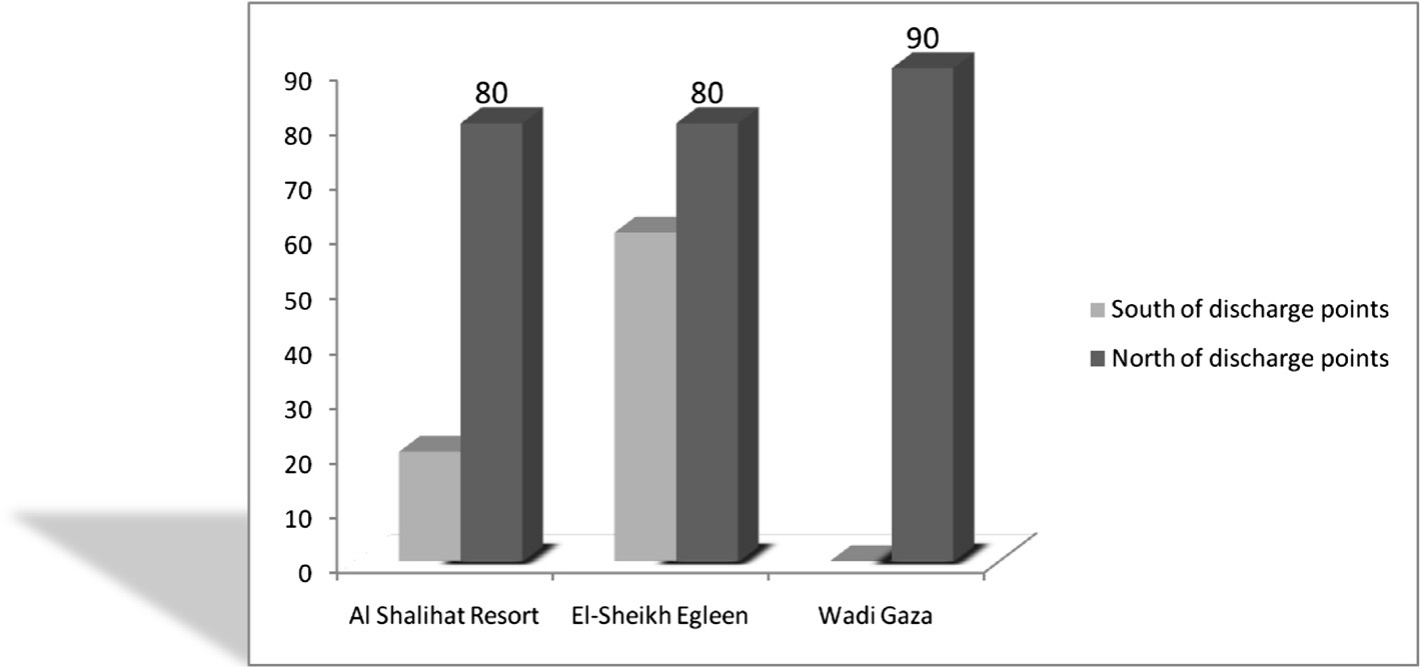
Mean of parasitic contamination in North and South of the discharge points.

Figure 4 shows the mean of parasitic contamination for the six samples taken from the southern side of each of the major discharge points (wastewater sources) compared with that of the other six samples taken from the northern side of those discharge points. The results show that all of the samples located in the northern side of the discharge points have a higher level of parasitic contamination than the samples from the southern side of the discharge points.

According to Figure 4, the percentage of the parasitic contamination in the northern side of Wadi Gaza (source of raw sewage) was 90% compared to zero contamination in the southern side of the valley, while the percentage of the parasitic contamination in the northern side of El-Sheikh Egleen (outlet of GWWTP) was 80% compared to 60% contamination in the southern side of this outlet point. Also, the percentage of the parasitic contamination in the northern side of Al Shalihat resort (outlet of sewage) was 80% compared to 20% contamination in the southern side of this outlet point. These results show that the seawater southern westerly currents have a significant effect on the dispersion of the sewage contaminants (including the parasites) along the Gaza City seashore.

## Discussion

Recreational seawater polluted by faecal discharges from wastewater discharge points may transport a variety of human pathogenic microorganisms. Because the detection of all waterborne potential faecal pathogens is very sensitive, various indicators of faecal contamination are usually used to detect faecal pollutions in natural waters [20].

### Contamination of seawater

In the Gaza Strip, population growth rate is very high (4.8 percent per annum), it means that about 75,000 inhabitants are added to the strip each year, increasing the population density and adding a load on the environment. In the Gaza Strip, about 69 MLD of partially treated sewage is discharged into the sea and so is 20 MLD of raw sewage [21]. This wastewater reaches the beach through many ways with several flow rates. The huge amount of the wastewater generated from the Gaza Strip is from Gaza City and it is considered as the main source of pollution in the shoreline region. Several sewage outlets have been discovered along the shoreline region of the Gaza City, the main outlets discharging raw sewage into the seashore region were Wadi Gaza, El-Sheikh Egleen and nearby Al Shalehat Resort. Hundreds of thousands of people in the Middle Area are living without a sanitary sewage collection system and without a treatment plant, 45% of them rely on discharging their raw sewage directly into Wadi Gaza and ultimately to the seashore region. Other thousands of cubic meters of partially treated wastewater are discharged directly into the seashore at zone (B), and a hundred cubic meters of raw sewage discharged nearby Al Shalehat Resort at zone (D). Continued dependence on ageing and poor cesspits, septic tanks and drain fields (or other on-site sewage disposal systems, OSDS) in coastal regions might cause excess loading of nutrients and microbes into seawaters [22]. The second identified outlet after Wadi Gaza was Al Sheikh Ejleen wastewater treatment plant discharge point, which discharges a huge amount of partially treated (sometimes untreated) wastewater produced from the central WWTP of Gaza City; the receiving area of this outlet on the seashore was zone (B). The beach at this region was contaminated to a high rate with parasites. The third outlet was nearby Al Shalehat resort, and in spite of the low flow rate of the concentrated sewage discharged through it, a high level of parasitic contamination was found nearby this outlet (zone D). This may be attributed to the continuous discharge of raw and polluted wastewater through this outlet. Because the sewage discharge points were far away from zones C and E, it makes these zones lower in parasite prevalence and contamination than zones A, B and D. Because there are no sewage outlets and the fishers marina tongue acts as a barrier to the distribution of parasites along the shoreline for long distances from the outlets toward the northern side of the study area, no parasitic contamination has been detected in the seawater of zone F, which is located behind the fishers marina in the opposite side of the carrying currents. In the current study, 52 seawater samples were analyzed in order to examine the parasitic contamination. The findings of the analyses showed that the contamination of the seawater in the Gaza City seashore region is relatively very high, 48.1% of the entire study area was contaminated with parasites due to the huge amount of raw and partially treated sewage that is discharged in the shoreline region of Gaza City as mentioned previously. In addition to faecal indicator organisms, enteric pathogens such as viruses and protozoa are found at high levels in human faeces and these may also contaminate coastal recreational and shellfish harvesting waters [23]. These parasites (pathogens) may cause infection through incidental ingestion of environmental waters during recreational bathing. The current study has used the presence of the gastrointestinal parasites as indicators of the seawater pollution, parasites survive longer than the indicator bacteria in coastal waters, and their survival may still be enhanced at low water temperatures [24]. The occurrence of parasites in the winter months may also be related to seasonal cycles in infection and excretion in the population [25]. The current study has used parasites as an indicator of the pollution in order to evaluate the status of the seashore region of Gaza City. There are several inherent disadvantages to using fecal coliforms as indicators of wastewater contamination of seawater in tropical and subtropical regions [26]. The current study used an Acid-fast stain for the detection of *Cryptosporidium parvum*; it is a protozoan that produces an unpleasant gastric and diarrheal illness known as cryptosporidiosis. It is able to survive extended periods under harsh environmental conditions [27]. *Cryptosporidium*, therefore, can cause some alarming public health problems, particularly for people with weakened immune systems, especially Acquired Immunodeficiency Syndrome (AIDS) patients, in whom severe and protracted diarrhea can persist for months with considerable weight loss and mortality [28]. In the present study, the protozoan parasite that has been detected in the seawater of the beach along the shoreline region of Gaza City was *Giardia lamblia*; it is the most common cause of human protozoan infection [29]. *Giardia lamblia* was reported in many studies in the Gaza Strip [30]. The relatively low percentage of parasites in the seawater in the current study may be attributed to the sedimentation impact which affects the concentration of the parasites in the upper layer of the seawater column (seawater sampling layer). Reduction of protozoan parasites (*Giardia* and *Cryptosporidium*) observed in seawater [31] was thought to be due to the sedimentation of parasites to the bottom of the water column. Thus, the bottom sediments of the polluted seawater could potentially serve as a reservoir of human pathogens, which could be released into the water column by storms (waves and agitated tidal movements) or manmade events [32]. In the present study, sampling processes have been carried out in the first hours of the day (morning), with no waves and agitation movements, all of that may affect the prevalence of parasites and finally the detection of parasites and the diagnostic process. Sedimentation is one of the many processes, which may be involved in the reduction of pathogens in seawater [33]. The current study used a modified Ziehl-Neelsen staining technique in order to increase the accuracy in the analysis process. This technique is restricted for the *Cryptosporidium spp*. and *Cyclospora.* Tuli *et al*., [34], found that the Modified Ziehl-Neelsen technique (Acid-fast stain) is better for detecting *Cryptosporidium spp*. compared to Safranin staining. Kehl *et al.*, [35], reported that the Modified Ziehl-Neelsen staining is 96% sensitive and 99% specific for the detection of *Cryptosporidium spp.*.

### Variables affecting the prevalence and distribution of the parasites in the shoreline region

The current study also found that the distance of the sewage discharge points along the shoreline region are inversely proportional to the prevalence of human gastrointestinal parasites. Accordingly, the zones most contaminated with parasites in the entire study area throughout the current research were those most adjacent to the sewage outlets, such as zones (A, B and D) which received raw sewage from Wadi Gaza which was heavily polluted by very concentrated raw sewage, Gaza WWTP discharge point and nearby Al Shalihat resort outlet respectively. The current study found that zones (A, B and D) were the most contaminated zones with parasites, while the uncontaminated zones were those distant from the wastewater outlets such as zone F. These findings have been supported by Savage, [36] and Daskin *et al*., [37], where they found that the distance from a wastewater source may reduce the concentration of the nutrients (Nitrogen and Phosphorus) and microbes as they are diluted by receiving seawater. In the current study, it is clear that the outlet with a high flow rate makes the adjacent zone more polluted and for a long distance, while the outlets with a low flow rate contaminate the seawater to a lower level. These findings were comparable with other studies. Flow rate variation in wastewater discharge may also have affected the total faecal coliform and parasite load to receiving seawaters because increasing the wastewater flow rate leads to an increase in the pollution and parasitic contamination of the seawater [38]. Also, the current study illustrates that the shoreline region was influenced by carrying currents, its direction from the south to the north. It affects the distribution of the parasite contamination for a certain distance. Figure 3, shows the mean of parasitic contamination for the six samples taken from the southern side of each of the major discharge points (wastewater sources) compared with that of the other six samples taken from the northern side of those discharge points. The results show that all of the samples located in the northern side of the discharge points have a higher level of parasitic contamination than the samples from the southern side of the discharge points. These results show that the seawater south westerly currents have a significant effect on the dispersion of the sewage contaminants (including the parasites) along the Gaza City seashore.

## Conclusion

The observations of the present study revealed a high level of parasitic pollution in most of the points which were investigated along the Gaza City coast line, as indicated by the high prevalence of human gastrointestinal parasites. Pollution was due to the direct disposal of wastewater into seawater and beach sand without treatment or with partial treatment.

### Recommendations

It is recommended that effluent quality should be improved by constructing a sufficient and efficient new WWTP and rehabilitating and upgrading the old WWTP. The public should be informed clearly by posting signs indicating polluted areas. A Health education program should be established at primary and secondary level to enhance public awareness about this issue and warn the public about the dangers of swimming in contaminated areas through the media.

## Competing interests

The authors declare that they have no competing interests.

## Authors’ contributions

AHH: participated in the design of the study, collection of data and performed the statistical analysis.AIAH: shared in the design, interpretation of the results, draft of the manuscript and preparation for submission for publication. YAAS participated in its design and coordination and helped to draft the manuscript. All authors read and approved the final manuscript.
